# Combined Yttrium-90 microsphere selective internal radiation therapy and external beam radiotherapy in patients with hepatocellular carcinoma: From clinical aspects to dosimetry

**DOI:** 10.1371/journal.pone.0190098

**Published:** 2018-01-02

**Authors:** Ti-Hao Wang, Pin-I Huang, Yu-Wen Hu, Ko-Han Lin, Ching-Sheng Liu, Yi-Yang Lin, Chien-An Liu, Hsiou-Shan Tseng, Yu-Ming Liu, Rheun-Chuan Lee

**Affiliations:** 1 Department of Radiation Oncology, China Medical University Hospital, Taichung, Taiwan; 2 Department of Oncology, Division of Radiation Oncology, Taipei Veterans General Hospital, Taipei, Taiwan; 3 Faculty of Medicine, School of Medicine, National Yang-Ming University, Taipei, Taiwan; 4 Institue of Public Health, School of Medicine, National Yang-Ming University, Taipei, Taiwan; 5 Department of Nuclear Medicine, Taipei Veterans General Hospital, Taipei, Taiwan; 6 Department of Radiology, Taipei Veterans General Hospital, Taipei, Taiwan; 7 Institue of Traditional Medicine, School of Medicine, National Yang-Ming University, Taipei, Taiwan; Chang Gung Memorial Hospital Kaohsiung Branch, TAIWAN

## Abstract

**Purpose:**

Selective internal radiation therapy (SIRT) is an effective treatment strategy for unresectable hepatocellular carcinoma (HCC) patients. However, the prognoses of patients with portal vein thrombosis, extra-hepatic metastases, or residual tumors remain poor when treated with SIRT alone. In these patients, sequential external beam radiotherapy (EBRT) may offer a chance of salvage. Here, we reported the clinical outcomes and the detailed dosimetry analysis of 22 patients treated with combination therapy.

**Methods:**

Between October 2011 and May 2015, 22 consecutive patients who underwent EBRT after yttrium-90 (^90^Y) SIRT were included in this study. The post-SIRT ^90^Y bremsstrahlung SPECT/CT of each patient was transferred to dose distribution by adopting the local deposition hypothesis. The patient-specific 3-dimensional biological effective dose distribution of combined SIRT and EBRT was generated. The overall survival and safety were evaluated. The relationship between dosimetric parameters and liver toxicity was analyzed.

**Results:**

The mean administered activity of SIRT was 1.50 GBq (range: 0.5–2.8). The mean prescribed dose of EBRT was 42.3 Gy (range: 15–63) in 14 fractions (range: 5–15) and was targeted to the residual liver tumor in 12 patients (55%), portal vein thrombosis in 11 patients (50%), and perihilar lymphadenopathies in 4 patients (18%). The overall 1-, 2-, and 3-year survival rates were 59.8%, 47.9%, and 47.9%, respectively. Overall, 8 patients (36%) developed > grade 2 liver toxicities, and the Child-Pugh score prior to EBRT strongly affected the toxicity risk. A dosimetry analysis restricted to 18 Child-Pugh A/B patients showed that the V100 (The fraction of normal liver exposed to more than 100 Gy) to V140 significance differed between patients who did or did not experience hepatotoxicity. The V110 was the strongest predictor of hepatotoxicity (18.6±11.6% vs 29.5±5.8%; P = 0.030).

**Conclusion:**

Combined therapy is feasible and safe if patients are carefully selected. Specifically, 3-dimensional dosimetry is crucial for the evaluation of efficacy and toxicity. The normal liver V100 to V140 values of the combined dose should be as low as possible to minimize the risk of liver toxicity.

## Introduction

Selective internal radiation therapy (SIRT) is an effective local treatment in hepatocellular carcinoma (HCC) patients not eligible for surgery[1–2]. However, the presence of portal vein thrombosis (PVT), extra-hepatic lymphadenopathy, or residual tumor remains a poor prognostic factor after SIRT. In these circumstances, the combination of external beam radiotherapy (EBRT) is reasonable and may improve tumor control and survival. Meanwhile, liver is a radiosensitive organ, which makes liver toxicity a major concern in combined treatment[3]. To minimize liver toxicity associated with combined treatment, a precise radiation dose distribution of both treatments is crucial. Unfortunately, the dosimetric methods currently used in SIRT can only estimate the average tumor and normal liver dose despite its highly nonuniform distribution nature. Nevertheless, a delicate physical and biologic conversion yielded from a post-SIRT image can be used as a source of patient-specific 3-dimensional voxel-based dose reconstruction. These methods have gained popularity and have been applied to evaluate outcome in recent years[4].

In the present study, we reported the efficacy and safety of combined SIRT and EBRT in HCC patients. To this end, we generated 3-dimensional voxel-based biologic equivalent dose (BED) distributions for each patient, and the dosimetric parameters of SIRT, EBRT, and combined treatment were analyzed to identify the dose-volume parameters predictive of hepatotoxicity.

## Materials & methods

### Patient characteristics

Between October 2011 and May 2015, a total of 95 consecutive unresectable HCC patients were treated with yttrium-90 (^90^Y) SIRT using resin microspheres (SIR-spheres; Sirtex, Lane Cove, Australia). Of these patients, 22 underwent liver EBRT after SIRT. Two patients received 2 courses of SIRT (one received sequential treatment for bi-lobar disease), and 5 patients received 2 courses of radiotherapy. The present retrospective study was approved by the institutional review board (No. 2012-02-009AC).

### Y-90 treatment

In our institute, SIRT was performed according to consensus recommendations. Each candidate was discussed by a multidisciplinary tumor board that included intervention radiologists, nuclear medicine specialists, and radiation oncologists. An angiographic evaluation of the hepatic vasculature and embolization of parasitized or variant arteries was conducted 1 week prior to treatment. A pretreatment ^99m^Tc macro-aggregated albumin (MAA) scan was performed to evaluate lung and gastrointestinal tract shunt fractions. This treatment was contraindicated in patients with a shunt fraction of more than 15%. An experienced intervention radiologist performed the entire procedure, and the injected dose was calculated with the partition model[5] and approved by the multidisciplinary tumor board. All prescribed activities of ^90^Y were recalibrated on the day of treatment. After treatment, the patient was transferred to undergo ^90^Y bremsstrahlung SPECT/CT in order to acquire the radiopharmaceutical distribution. Bremsstrahlung SPECT/CT was performed on the same scanner as 99mTc-MAA SPECT/CT. Three energy window widths of 20% centered at 70 keV, 135 keV, and 167 keV were defined, and a 128×128 matrix with 60 frames (30 s per frame) was used for SPECT acquisitions. The attenuation of images was corrected by CT, and the images were reconstructed using an OSEM algorithm with two iterations and 10 subsets. The resolution of SPECT was 128 x 128, with square pixels of 4.42 mm. The scan parameters for CT were 120 kV and smart mA with 3.75-mm slices. However due to data missing, SPECT/CT were available from only 20 patients and used for dose analysis.

### EBRT treatment

During the CT simulation and treatment, the patients were fixed with a body vacuum-bag. An abdominal compressor and/or four-dimensional computed tomography (4D CT) were used as methods of respiratory control, and the radiotherapy plan and calculations were performed with the Eclipse system software (Varian Medical Systems, Palo Alto, CA) or helical tomotherapy (HT) (Accuray, Sunnyvale, CA). Intensity-modulated radiation therapy (IMRT) or Intensity modulated arc therapy (IMAT) techniques were used in the majority of patients.

The extent of gross tumor volume (GTV) in EBRT may contained residual liver tumor and/or portal vein thrombosis and/or perihilar lymphadenopathies, by radiation oncologist’ judgement. The residual liver tumor was defnined by tri-phase CT or contrast-enhanced MRI post SIRT treatment. I’ts possible that viable tumor whould not be fully covered by EBRT due to balance of efficacy and toxicities. After delineation, a 5-10mm symetric expansion were made for clinical target volume (CTV), and another 5mm symetric expansion were made for planning target volume (PTV). Considering previous SIRT treatment, we used tight constraints for liver during EBRT: The mean dose of normal liver less than 20Gy, V5 (The fraction of normal liver exposed to more than 5 Gy) <86%, V10<68%, V20<49%, V30<28%, V40<20%[6–8]

### Voxel method of SIRT

To simplify the calculation and optimize it for clinical use, we made several assumptions. First, we assumed that the ^90^Y particles are permanently trapped in the microvasculature and that the deposition distribution is identical to the post-treatment bremsstrahlung SPECT/CT. Thus, the voxel and total activity of ^90^Y was proportional to the voxel and total counts of SPECT, respectively. Based on the work of Chiesa et al.[9], we assumed local energy deposition, i.e., ^90^Y only self-irradiates within each voxel. For each voxel *i*, *A(*^*90*^*Y)* is the activity of ^90^Y, *C*_*i*_ is the count of voxel *i* presented in SPECT/CT. *C*_*total*_ is the total voxel count presented in SPECT/CT. The absorbed dose of voxel *i,* *D*_*i*_ can be calculated as follows:

$$D_{i}=A({}^{90}Y)\frac{50Gy/[GBq]1kg}{{\left(4.42\;mm\right)}^{3}\times 1g/{cm}^{3}}\times \frac{C_{i}}{C_{total}}$$

### BED conversion

In the next step, the voxel physical dose was transformed into the voxel BED. Because SIRT is a low-dose-rate brachytherapy, time factors should be considered in BED transformation. For a voxel *i*, *D*_*i*_ is the total radiation dose, and *FD*_*i*_ is the fraction dose in EBRT. Thus, BED can be calculated as follows:

ForEBRT:BEDi=Di(1+FDiαβ)

ForSIRT:BEDi=Di(1+Di×Trepαβ×(Trep+Teff))

The following radiobiology parameters were employed[10]: *T*_*eff*_ = 64.2 hours and *T*_*rep*_ = 2.5 hours for a normal liver and 1.5 hours for a tumor; the αβ ratio can differentiate early- and late-responding tissue and is typically 2.5 Gy for a normal liver, and 10 Gy for a tumor. BED-maps for both SIRT and EBRT were generated using an in-house code in Matlab (2013b, Mathworks, Natick, MA).

### Dose summation

Finally, the CT simulation was registered to the CT from the bremsstrahlung scan using rigid registration with the Eclipse system software. BED-maps of SIRT and EBRT were summed voxel-by-voxel according to the registration relationship. The normal liver and tumor were segmented in simulation CT. The cumulative BED histogram of combined treatments was computed for each patient.

### Clinical data evaluation

Follow-up, including a physical examination and laboratory testing, was performed for all patients each month for three months after treatment, then at 3-month intervals thereafter. Serum liver function tests, including total serum bilirubin, aspartate aminotransferase (AST), alanine aminotransferase (ALT), alkaline phosphatase (ALP), gamma-glutamyl transferase (GGT), and albumin measurements, and the Child-Pugh score were obtained pre-SIRT and pre-EBRT (baseline) and 2, 4, and 8 weeks after EBRT treatment. An image study was usually performed every 3 months. The radiation-related hepatic toxicities were recorded within 6 months of EBRT and were graded based on the Common Terminology Criteria for Adverse Events (CTCAE) of the National Cancer Institute, version 4.03[11]. The specific toxicity grading table was listed in S1 Table.

### Response evaluation

Although mRECIST had better response evalaution on Sorafenib or Transarterial Chemoembolization (TACE), it has not been verified in evaluating HCC post SIRT treatment. In a recent study, the reserchers found it is common that tumor borders exhibit a pseudonodular area of enhancement, or that intratumoral enhancing septa are being observed after SIRT treatment, which made mRECIST a difficult assessment in evaluating tumor.

We used the World Health Organization (WHO) tumor response criteria[12] to evaluate tumor response. The response rate was graded as follows: complete response (CR), 100% decrease in the sum of cross products; partial response (PR), more than 50% decrease in the sum of cross products; and progressive disease, more than 25% increase in the sum of the cross products. All other responses were defined as stable disease.

### Statistics

Actuarial estimates of survival are displayed as Kaplan–Meier plots. The dosimetric parameters were compared between patients with/without > grade 2 toxicities using Student’s t-test. The following dosimetric parameters were analyzed: the mean BED to the normal liver (Dmean) and the V10 (percentage of normal liver volume that received > 10Gy BED) to V180 values at increments of 10 Gy. All statistical analyses were conducted with R (version 3.1.2)[13]. A BED analysis was carried out using the R “DVHmetrics” package.

## Results

### Patient characteristics and treatments

The median age of our patients at the time of SIRT was 59 years (IQR: 49–63). Eighteen patients (82%) were male, and all patients had a Grade 0 or 1 ECOG performance status. Prior to SIRT, only 4 (18%) patients were therapy-naive; the remaining patients had received intensive therapy, including surgical resection, radiofrequency ablation (RFA), trans-arterial chemo-embolization (TACE), and chemotherapy. Seventeen patients (77%) had chronic hepatitis B infections, 2 patients had chronic hepatitis C infections, and 2 patients had both hepatitis B and C infections. Four patients (18%) had ECOG 0 status, sixteen patients (72%) had ECOG 1 status, and two patients (10%) had ECOG 2 status before EBRT. All patients exhibited Child-Pugh A liver cirrhosis prior to SIRT, and the mean tumor size was 7.3 cm (range: 2.2–19 cm).

Nineteen of 22 patients (86%) had undergone SIRT for segmental treatment, and 3 patients (14%) had received whole liver treatment. The mean administered activity of SIRT was 1.50 GBq (range: 0.5–2.8 GBq), and the mean interval between SIRT and EBRT was 214 days (range: 4–1181 days). Prior to EBRT, seventeen patients (77%) had Child-Pugh A liver cirrhosis, 3 patients (14%) had Child-Pugh B liver cirrhosis, and 2 patients (9%) had Child-Pugh C liver cirrhosis. The mean prescribed dose of EBRT was 42.3 Gy (range: 15–63 Gy) in 14 fractions (range: 5–15). The target of EBRT contained residual liver tumor in 12 patients (55%), portal vein thrombosis in 11 patients (50%), and perihilar lymphadenopathies in 4 patients (18%).

A voxel-based dosimetry showed that the mean and minimal tumor absorbed doses of SIRT were 115.8 Gy (range: 37.1–245.5 Gy) and 60.1 Gy (range: 11.6–184.5 Gy), respectively. The mean normal absorbed dose in the liver was 39.6 Gy (range: 9.8–68.6 Gy). In 11 patients with PVT, the mean and minimal SIRT doses absorbed by the PVT were 66.2 Gy (range: 46.4–83.0 Gy) and 26.9 Gy (range: 12.0–38.5 Gy), respectively. Three patients received an incomplete radiotherapy course due to their critical status. The actual delivered mean target and normal liver absorbed dose of EBRT were 42.3 Gy (range: 15–63 Gy) and 11.7 Gy (range: 5.0–18.2 Gy), respectively.

The median follow-up time after SIRT for all patients was 340 days (range: 59–1645 days), and the demographic characteristics, tumor factors, base-line liver function, and treatments of the study cohorts are summarized in Table 1.

**Table 1:** Demographic characteristics, tumor factors, base-line liver function, and treatments of the study cohorts.

Characteristics	N = or mean (range)	%
Median Age, year (range)	59 (33–83)	
> = 65	5	22.7
<65	17	77.3
Sex(M)	18	81.8
Viral hepatitis		
None	2	9.1
HBV	17	77.3
HCV	4^a^	18.2
ECOG		
0	4	18
1	16	72
2	2	10
Prior therapy		
None	4	18.2
Resection	7	31.8
RFA	8	36.4
TACE	13	59.1
Sorafenib	3	13.6
Thalidomide	3	13.6
Child-Pugh before SIRT		
A	22	100
B	0	0
Child- Pugh before EBRT		
A	17	77.3
B	3	13.6
C	2	9.1
Tumor size, cm (range)	7.3 (2.2–19)	
Multiple	12	54.5
Liver volume, mL (range)	1594 (1169–2824)	
Portal vein invasion		
None	4	18.2
Main trunk	9	40.9
Lobar branch	6	27.3
Segmental branch	3	13.6
SIRT Treatment factors		
SIRT dose, GBq (range)	1.5 (0.5–2.8)	
Whole liver treatment	3	13.6
Segmental treatment	19	86.4
Mean tumor dose, Gy (range)	115.8 (37.1–245.5)	
Minimal tumor dose, Gy (range)	60.1 (11.6–184.5)	
Mean normal liver dose, Gy (range)	39.6 (9.8–68.6)	
SIRT and EBRT time interval, days (range)	214 (4–1181)	
EBRT target		
Residual tumor	12	54.5
Portal vein thrombosis	11	50
Lymphadenopathy	4	18.2
EBRT target dose, Gy (range)	42.3 (15–63)	
EBRT liver dose, Gy (range)	11.7 (5–18.2)	

^a^ includes two patients with co-infection of hepatitis B. Abbreviation: HBV, hepatitis B virus; HCV, hepatitis C virus; ECOG, Eastern Cooperative Oncology Group; RFA, Radiofrequency ablation; TACE, Transcatheter arterial chemoembolization; SIRT, Selective internal radiation therapy; EBRT, external beam radiotherapy.

### Efficacy

**Fig 1:**
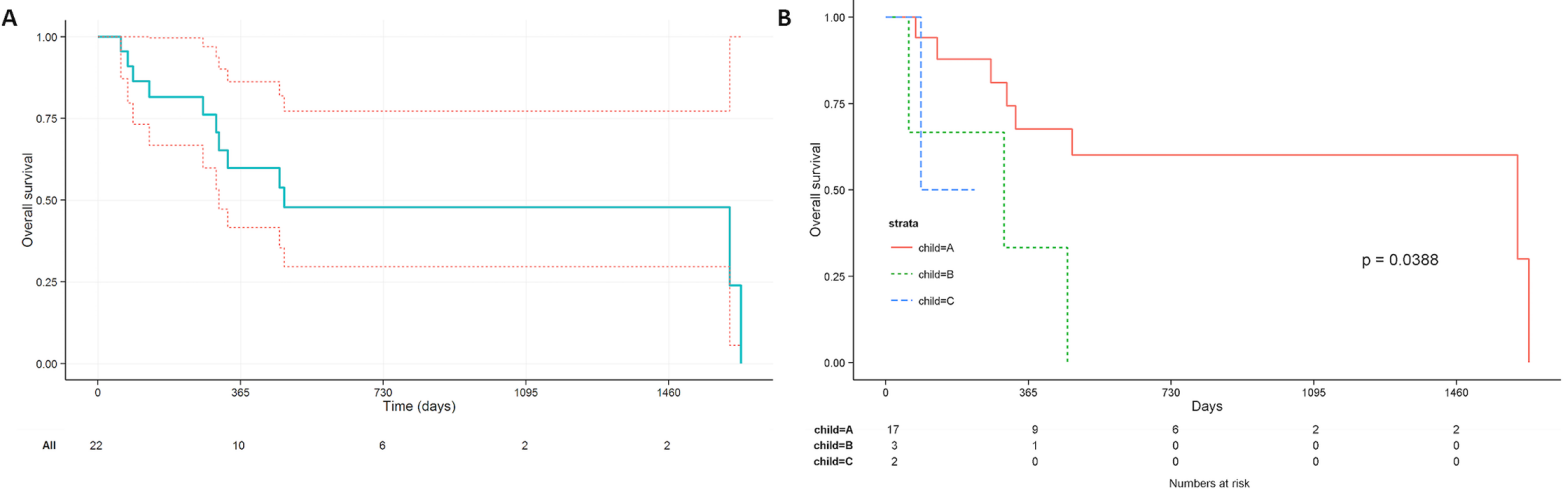
The overall survival of patients. (A)The overall survival of patients treated with combined therapy. Dashed lines indicate the confidence interval. (B) The overall survival of patients treated with combined therapy, stratified by Child-Pugh score before external beam.

Survival was assessed starting on the day of first SIRT treatment, and the Kaplan-Meier plot is shown in Fig 1. The 1-, 2-, and 3-year overall survival at were 59.8%, 47.9%, and 47.9%, respectively. The median survival was 477 days (95%CI: 310-infinity days). The median survival was significantly better in patients with Child A disease prior to EBRT (median 1616 days, 95%CI: 333-infinity days) than in patients with Child B or C disease (median 303 days, 95%CI: 90-infinity days, p = 0.01). The image study three months after EBRT showed primary tumor partial regression in 5 patients (23%) and complete remission in 4 patients (18%). Six patients (27%) exhibited primary disease progression after treatment, and evidence of new liver lesions developed in 7 patients (32%) during follow up. In 11 patients with PVT, six patients (55%) exhibited thrombosis improvement after EBRT. At the time of analysis, 12 patients had died, 7 of whom died as a result of primary or metastatic disease progression, whereas 5 patients died as a result of parenchymal liver failure from either disease burden or treatment complications.

### Safety

Overall, 8 patients (36%) developed > grade 2 liver toxicities. Five patients (23%) experienced grade 5 liver failure toxicity. Nevertheless, none of the patients developed liver failure in the absence of intrahepatic tumor progression. Of the patients with Child A, B, and C cirrhosis (N = 17, 3, 2) before EBRT, 4 patients (24%), 2 patients (67%), and 2 patients (100%) developed > grade 2 hepatic failure, respectively. Other than hepatic toxicities, one patient had small intestine perforation 6 months after EBRT and was successfully treated by surgery. The hepatic toxicities profile, stratified by cirrhosis severity was demonstrated in Table 2. Among 9 patients who had more than grade 2 hepatic enzymes increased, two patient had no HBV/HCV infection. Seven patients had chronic hepatitis B infection, and one patient had co-infection of hepatitis C. All patients received anti-viral treatment (lamivudin, adevofir, tenofovir, and baraclude) except one patient, who had persistent low serum HBV titers. In these 7 patients suffered from liver toxicities, only 2 patients had serum HBV DNA ≥10-fold the baseline level.

**Table 2:** The hepatic toxicities profile, stratified by Child-Pugh score before external beam radiotherapy.

	A (N = 17)	B (N = 3)	C (N = 2)
Hepatic enzymes increased			
1	8(47%)	1(33%)	0
2	5(29%)	1(33%)	1(50%)
3	0	1(33%)	1(50%)
Bilirubin increased			
1	4(24%)	0	0
2	1(6%)	1(33%)	0
3	0	1(33%)	1(50%)
4	4(24%)	1(33%)	1(50%)
Hepatic failure			
3	0	0	0
4	1(6%)	1(33%)	1(50%)
5	3(18%)	1(33%)	1(50%)

### Dosimetric analysis

**Fig 2:**
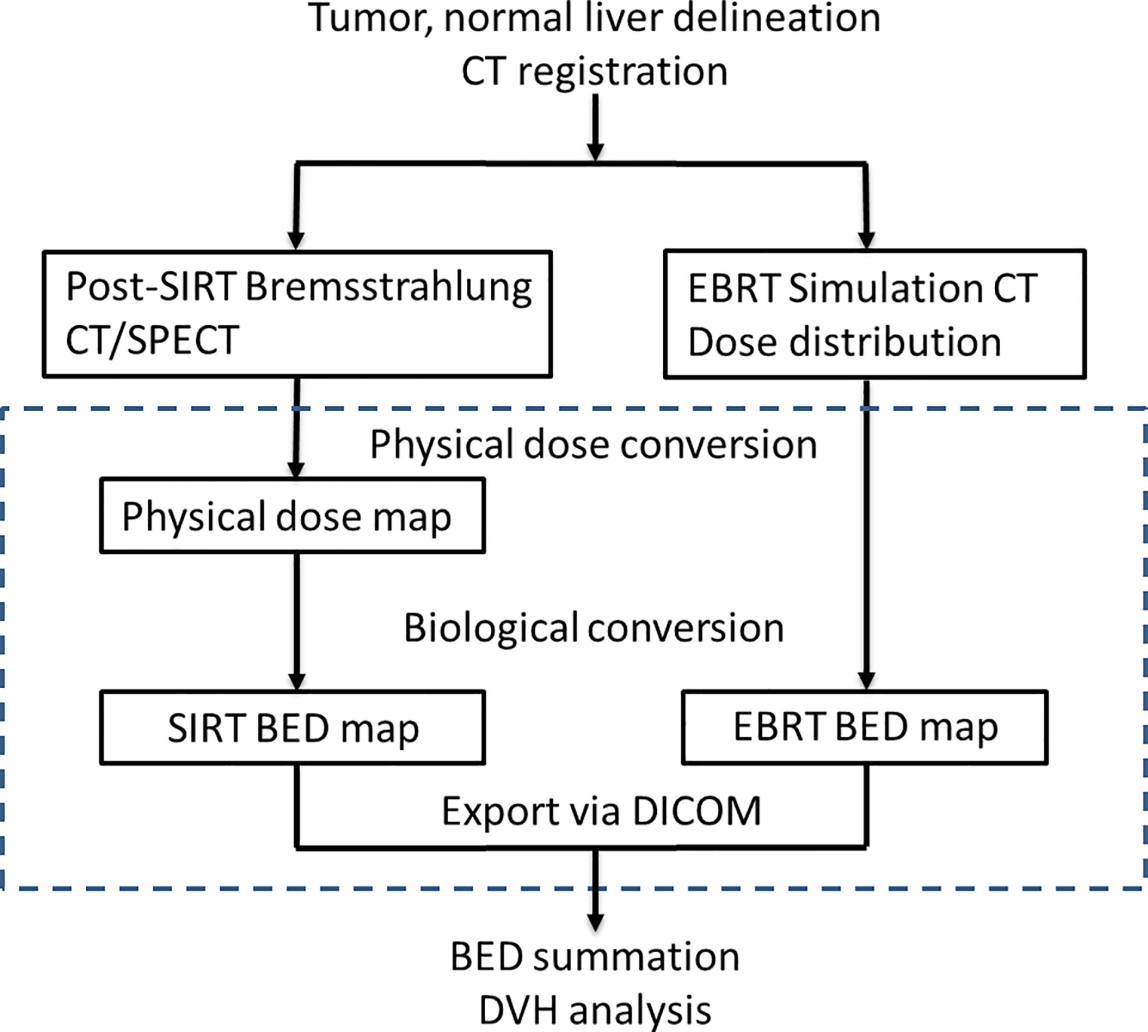
General workflow of SIRT and EBRT BED map summation. The blocks are the 3D data in DICOM format. The dashed line encloses the process done in Matlab. The process outside was done in Eclipse system. DICOM: Digital Imaging and Communications in Medicine.

**Fig 3:**
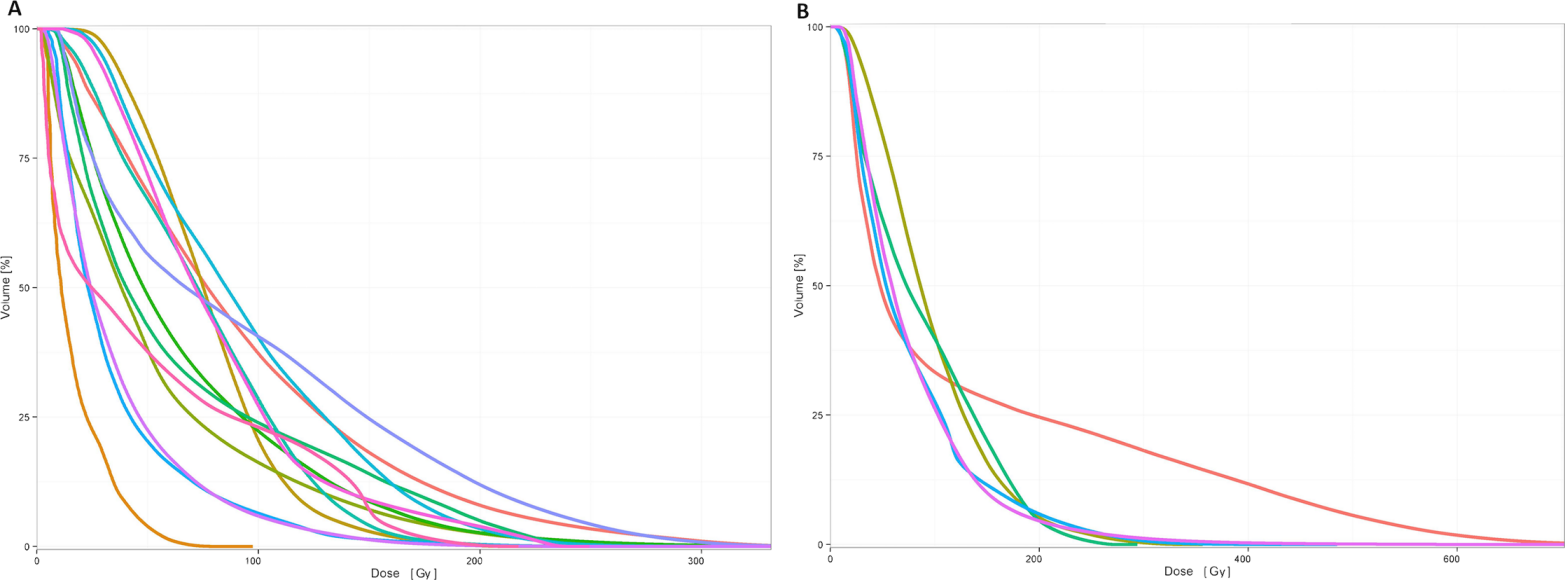
The summed biological equivalent dose volume histogram of normal liver. (A) patients with no > grade 2 liver toxicities. (B) patients with > grade 2 liver toxicities.

Fig 2 illustrates the general workflow of dose summation. Specifically, the 3-dimensional BED distribution of SIRT and EBRT were generated for each patient. The mean tumor target BEDs of SIRT and EBRT were 261.8 Gy (range: 58.6–388.3 Gy) and 96.2 Gy (range: 23.1–143.6 Gy), respectively. The mean normal liver BEDs of SIRT and EBRT were 65.4 Gy (range: 12.1–168.3 Gy) and 19.6 Gy (range: 7.1–38.0 Gy), respectively. Detailed BED table was listed in S2 Table. After adding the BED maps of both treatments, we analyzed the relationship between dosimetry and hepatic toxicity. The total dose volume histograms (DVH) of each patient are depicted in Fig 3. We only analyzed patients with Child A or B liver cirrhosis for whom SPECT/CT was available (N = 18) prior to EBRT. As shown is Table 3, all dosimetric parameters, from V100 (The fraction of normal liver exposed to more than 100 Gy) to V140, significantly differed between patients who did or did not develop hepatotoxicity. The V110 was the strongest predictor of hepatotoxicity (18.6±11.6% vs 29.5±5.8%; P = 0.030), but the mean summed dose delivered to the normal liver did not significantly differ between the two groups (65.5±24.1 Gy vs 92.6±23.9 Gy; P = 0.051). Notably, neither dosimetry for SIRT nor that for EBRT alone can better predict hepatic toxicity.

**Table 3:** Association of biological dosimetric parameters of SIRT, EBRT, and combined therapy and hepatic toxicities.

CTCAE >2 Hepatotoxicity									
	SIRT + EBRT			SIRT			EBRT		
	No (N = 10)	Yes (N = 5)	p value	No (N = 10)	Yes (N = 5)	p value	No (N = 10)	Yes (N = 5)	p value
V80 (%)	33.6(17.4)	42.1(7.6)	0.213	22.9(14.3)	31.3(4.1)	0.085	7(6.7)	8.8(2.9)	0.981
V90 (%)	27.7(14.6)	37.6(6.6)	0.095	18.0(13.5)	27.5(4.3)	0.050	5.3(6.6)	7.0(2.6)	0.964
V100 (%)	22.7(14.6)	33.5(5.9)	0.043	14.5(13.1)	24.2(4.8)	0.045	4.4(6.1)	5.3(2.4)	0.944
V110 (%)	18.6(11.6)	29.5(5.8)	0.030	12.0(12.4)	21.4(5.3)	0.052	3.5(5.8)	3.4(2.8)	0.582
V120 (%)	15.2(10.7)	25.4(6.4)	0.043	10.1(11.7)	18.9(5.7)	0.066	2.9(5.3)	2.6(2.4)	0.498
V130 (%)	12.5(9.7)	22.2(6.4)	0.047	8.7(10.8)	16.6(6.1)	0.093	2.2(4.6)	1.6(1.6)	0.331
V140 (%)	10.2(8.5)	19.5(6.4)	0.049	7.6(10.1)	14.5(6.5)	0.145	1.6(3.5)	1(1.4)	0.305
V150 (%)	8.0(7.5)	17.1(6.6)	0.051	6.7(9.4)	12.5(6.9)	0.223	0.6(1.6)	0.8(1.2)	0.362
V160 (%)	6.4(6.6)	15.1(6.7)	0.063	6.0(8.8)	10.6(7.4)	0.332	0(0)	0.5(0.9)	0.825
Dmean(Gy)	65.5(24.1)	92.6(23.9)	0.051	61.5(38.0)	75.7(22.1)	0.58	19.3(9.6)	20.5(3.7)	0.972

Values are shown as mean (standard deviation). Abbreviation: CTCAE: Common Terminology Criteria for Adverse Events, V80: the fraction of normal liver had received more than 80 Gy biological effective dose.

## Discussion

### SIRT 3-dimensional dosimetry

The dosimetry of radionuclide therapies is best depicted by patient-specific 3-dimensional imaging–based internal dosimetry, which has been applied to several cancer treatments[14–15]. Nevertheless, 3-dimensional dosimetry was not widely implemented in SIRT until recently[9–16], and SIRT is often planned using empirical models[5]. Although the distribution of ^90^Y microspheres is highly non-uniform, these empirical models can only estimate the “average” dose delivered to the tumor and normal liver, which could be misleading. For example, we found that the mean tumor dose of SIRT exceeded 100 Gy in most patients, whereas the minimal tumor dose was only 60 Gy. Using empirical models, this under-dosed area may be totally neglected by the “average” dose but may serve as a recurrence nidus. For this reason, 3-dimensional dosimetry should be used for both treatment planning and response evaluation. Garin et al. first reported the successful prediction of the tumor response based on a tumor dose calculated using a quantitative analysis of the ^99m^Tc-macroaggregated albumin SPECT/CT exam[17]. To meet clinical needs, Kalantzis et al. developed a user-friendly computational tool for patient-specific dosimetry[18].

Another benefit of 3-dimensional dosimetry is that it can be easily incorporated into models inherited from external-beam radiation therapy (EBRT). The clinical experience of EBRT is the most important reference for SIRT, and the DVH concept used in EBRT can be introduced to SIRT. Thus, the tumor control probability (TCP) and normal tissue complication probability (NTCP) models can be applied as they are in EBRT[19]. In fact, Strigari et al. implemented a TCP model of 73 HCC patients in SIRT[20], whereas an NTCP model for the liver has not yet been proposed.

### Efficacy and safety of combined therapy

The combination of EBRT with targeting radionuclide therapies has been tested in unresectable meningioma, brain metastasis, and breast cancer patients but not yet in HCC patients[21–23]. There are several advantages of this approach. First, the tumor area under-dosed by SIRT could be salvage by EBRT. Second, extra-hepatic lymphadenopathies are only targeted by EBRT but not SIRT. Finally, EBRT is more efficient in treating portal vein thrombosis (PVT) than SIRT and can result in a response rate as high as 50%[24]. Nakazawa et al. compared sorafenib therapy to radiotherapy in HCC patients with main or first-branch PVT, and they reported a significantly longer median overall survival in the EBRT group[25]. Although PVT is not contraindicated to SIRT, the reported median survival of patients who received SIRT alone was 6 months, and further declined to only 2.6 months if extra-hepatic metastases were present[2]. In our dosimetric statistics, the mean absorbed dose of portal vein thromboses by SIRT was only 60 Gy, indicating that, at least partly, SIRT has a modest effect on PVT. Thus, EBRT offers a salvage treatment option for these patients after SIRT. In our cohort, 12 patients with residual liver tumors, 4 with hilar lymphadenopathies, and 9 with main portal vein thromboses were treated with sequential EBRT. In these advanced HCC patients, the median survival was 16 months, which was better than the survival (10.7 months) of a comparable group in the SHARP trial[26]. Furthermore, the median survival in patients with Child A disease prior to EBRT exceeded 53 months. However, due to retrospective nature of the current study, the efficacy of this regimen should be validated in randomized controlled trials.

On the other hand, radiation-induced liver toxicity, which is related to the accumulated radiation dose in the normal liver, is a major concern associated with combined treatment. Radiation-induced liver disease (RILD), historically called “radiation hepatitis”, has long been reported in the EBRT literature as one of the most serious radiation-related complications. RILD typically develops 1–2 months after radiotherapy and is pathologically characterized as veno-occlusive disease. Its clinical findings are non-specific, including fatigue, abdominal pain, anicteric ascites, hepatomegaly, and abnormal liver function tests. Furthermore, the incidence of RILD varied across studies and was approximately 16–18% in HCC patients treated with 3D conformal radiation therapy[8]. Several reports have quantified the relationship between the radiation dose-volume and the risk of RILD[27]. Interestingly, SIRT, is associated with a distinct pattern of liver toxicities called radio-embolization-induced liver disease (REILD). This clinical syndrome consists of jaundice and ascites that appear 1–2 months after SIRT[28]. However, very few dosimetry analysis reports are currently available.

To our knowledge, only one report details the toxicity of EBRT combined with SIRT[29]. In this study, Lam et al. retrospectively studied 31 patients who underwent SIRT after previous EBRT. They analyzed the DVHs for EBRT, whereas the DVHs for SIRT were not considered. Patients who experienced hepatotoxicity received higher EBRT absorbed doses in the liver. The fraction of the liver exposed to at least 30 Gy (V30) was the strongest predictor of toxicity, with a threshold for hepatotoxicity at a volume of 13% and a threshold for fatal REILD at a volume of 30%. Notably, the liver was directly included in the radiation field in only 5 patients (16.1%) in the cohort. However, these data should be interpreted with caution. First, as marked by Cremonesi, the conclusions could be strongly influenced by the imbalance between the dosimetry information derived from EBRT and SIRT[30]. Second, a V30 threshold of less than 13% is almost impossible to achieve when planning hepatic EBRT. If this criterion holds true, it will eliminate the possibility of EBRT combination. Third, most of our patients (86%) were treated with selective segmental SIRT, whereas 84% of patients in Lam’s cohort[29] were treated with whole-liver SIRT, which may be associated with higher hepatic toxicity.

In toxicity analysis, we did not separate RILD from REILD because symptoms widely overlapped. Overall, grade 3 or higher hepatic toxicities developed in 36% patients, whereas 24% of patients with Child A disease developed hepatic toxicities. However, this toxicity rate may be overestimated because disease progression may have contributed to abnormal liver function. Considering the heavy treatment and fragile function of the liver in our patients, the obtained hepatic toxicity rate is acceptable. An accurate dose distribution is the key to investigate the dose-toxicity relationship. After analyzing the cumulative biological equivalent dose from EBRT, SIRT, and the combined therapy, we found that the combined BED can best predict hepatic toxicity. Among the dosimetric parameters, the V100Gy to V140Gy was significantly different between patients with/without hepatic toxicities. However, the cutoff tolerance level was not determined due to the limited number of cases.

### Strength and limitation

This study has three major contributions. First, we report a series of HCC patients who benefited from the combination of SIRT and EBRT. As a proof of concept, we demonstrated that the sum of the BED values of the two treatments accurately represented the delivered dose. Most importantly, we found that the sum of the dose parameters was significantly associated with liver toxicity.

Nevertheless, this study is subject to several limitations. First, the quantitative accuracy of the SIRT dose conversion from bremsstrahlung SPECT/CT is inferior to that of post-SIRT PET/CT[31]. Second, the local energy deposition assumption may oversimplify the actual dose distribution and can be replaced by Monte Carlo-based PDK in the future studies. The registration of SIRT and EBRT was another source of error, especially because the liver is a soft and mobile organ. Regarding biologic conversion, the LQ model, although extensively used in EBRT, has been criticized in the setting of high dose per fraction radiotherapy. Furthermore, other biological factors, such as viral hepatitis and time interval between SIRT and EBRT, were not included in the analysis due to small number of cases may play roles in the development of liver toxicities.

## Conclusion

In this study, we demonstrated the feasibility of combining SIRT and EBRT to treat HCC patients, and combined therapy may provide a survival benefit for carefully selected patients, especially patients with PVT. The toxicity rate was acceptable in patients with Child A cirrhosis prior to EBRT, and the combined BED distribution was valuable for predicting toxicity outcome. Overall, the most relevant dosimetric parameters were V100Gy to V140Gy. Future trials should be conducted to validate the combined treatment strategy guided by 3-dimensional SIRT dosimetry.

## Supporting information

S1 Table Hepatic toxicities grade according to CTCAE v4.03. (DOCX)

S2 Table Biological equivalent dose of SIRT and EBRT. (DOCX)
