# Supplementary material for: Combined Yttrium-90 microsphere selective internal radiation therapy and external beam radiotherapy in patients with hepatocellular carcinoma: From clinical aspects to dosimetry
Source: PLoS One. 2018 Jan 2;13(1):e0190098. doi: 10.1371/journal.pone.0190098 (PMC5749761; doi:10.1371/journal.pone.0190098)
Supplement: S1 Table — (DOCX) [file pone.0190098.s001.docx]

S1 Table. Hepatic toxicities grade according to CTCAE v4.03:

|  | Grade | | | | |
| --- | --- | --- | --- | --- | --- |
| Adverse Event | 1 | 2 | 3 | 4 | 5 |
| Alanine aminotransferase  increased | >ULN - 3.0 x ULN | >3.0 - 5.0 x ULN | >5.0 - 20.0 x ULN | >20.0 x ULN | - |
| Alkaline phosphatase  increased | >ULN - 2.5 x ULN | >2.5 - 5.0 x ULN | >5.0 - 20.0 x ULN | >20.0 x ULN | - |
| Aspartate aminotransferase  increased | >ULN - 3.0 x ULN | >3.0 - 5.0 x ULN | >5.0 - 20.0 x ULN | >20.0 x ULN | - |
| Blood bilirubin increased | >ULN - 1.5 x ULN | >1.5 - 3.0 x ULN | >3.0 - 10.0 x ULN | >10.0 x ULN | - |
| Hepatic failure | - | - | Asterixis; mild  encephalopathy; limiting self  care ADL | Moderate to severe  encephalopathy; coma; lifethreatening  consequences | Death |

CTCAE: Common Terminology Criteria for Adverse Events; ULN: Upper Limit of Normal; ADL: activities of daily living
