# Supplementary material for: Combined Yttrium-90 microsphere selective internal radiation therapy and external beam radiotherapy in patients with hepatocellular carcinoma: From clinical aspects to dosimetry
Source: PLoS One. 2018 Jan 2;13(1):e0190098. doi: 10.1371/journal.pone.0190098 (PMC5749761; doi:10.1371/journal.pone.0190098)
Supplement: S2 Table — (DOCX) [file pone.0190098.s002.docx]

S2 Table. Biological equivalent dose of SIRT and EBRT

|  | Mean Normal liver Dose | | Mean Tumor Dose | |
| --- | --- | --- | --- | --- |
| Patient | SIRT*(Gy) | EBRT**(Gy) | SIRT(Gy) | EBRT(Gy) |
| 1 | 68.85 | 17.85 | 168.99 | 134.77 |
| 2 | 71.02 | 8.34 | 311.72 | 50.22 |
| 3 | 54.97 | 16.82 | 488.47 | 93.02 |
| 4 | 68.14 | 26.53 | 251.72 | 23.17 |
| 5 | 59.21 | 12.29 | 215.45 | 131.37 |
| 6 | 12.12 | 10 | 58.64 | 35.38 |
| 7 | 60.21 | 17.58 | 241.9 | 93.95 |
| 8 | 46.96 | 7.1 | 274.26 | 38.27 |
| 9 | 39.61 | 28.62 | 222.74 | 120 |
| 10 | 58.67 | 23.2 | 289.29 | 136.93 |
| 11 | 70.6 | 22.34 | 267.59 | 96.51 |
| 12 | 33.36 | 22.05 | 278.93 | 128.63 |
| 13 | 65.37 | 22.98 | 377.84 | 86.09 |
| 14 | 87.81 | 5.69 | 237.45 | 71.52 |
| 15 | 168.33 | 17.88 | 388.32 | 116.61 |
| 16 | 51.49 | 29.88 | 221.65 | 136.38 |
| 17 | 16.34 | 38 | 156.47 | 143.35 |
| 18 | 57.17 | 18.4 | 437.48 | 111.44 |

*SIRT: Selective internal radiation therapy;

**EBRT: External beam radiotherapy
